# Reliability of prognostic biomarkers after prehospital extracorporeal cardiopulmonary resuscitation with target temperature management

**DOI:** 10.1186/s13049-021-00961-8

**Published:** 2021-10-09

**Authors:** Walter Petermichl, Alois Philipp, Karl-Anton Hiller, Maik Foltan, Bernhard Floerchinger, Bernhard Graf, Dirk Lunz

**Affiliations:** 1 Department of Anaesthesiology, University Hospital Regensburg, University of Regensburg Medical Center, Franz-Josef-Strauß-Allee 11, 93053 Regensburg, Germany; 2 Department of Cardiothoracic Surgery, University of Regensburg Medical Center, Franz-Josef-Strauß-Allee 11, 93053 Regensburg, Germany; 3 Department of Operative Dentistry and Periodontology, University of Regensburg Medical Center, Franz-Josef-Strauß-Allee 11, 93053 Regensburg, Germany

**Keywords:** Out-of-hospital cardiac arrest, Extracorporeal cardiopulmonary resuscitation, Target temperature management, Neurological outcome, Prehospital

## Abstract

**Background:**

Extracorporeal cardiopulmonary resuscitation (ECPR) performed at the emergency scene in out-of-hospital cardiac arrest (OHCA) can minimize low-flow time. Target temperature management (TTM) after cardiac arrest can improve neurological outcome. A combination of ECPR and TTM, both implemented as soon as possible on scene, appears to have promising results in OHCA. To date, it is still unknown whether the implementation of TTM and ECPR on scene affects the time course and value of neurological biomarkers.

**Methods:**

69 ECPR patients were examined in this study. Blood samples were collected between 1 and 72 h after ECPR and analyzed for S100, neuron-specific enolase (NSE), lactate, D-dimers and interleukin 6 (IL6). Cerebral performance category (CPC) scores were used to assess neurological outcome after ECPR upon hospital discharge. Resuscitation data were extracted from the Regensburg extracorporeal membrane oxygenation database and all data were analyzed by a statistician. The data were analyzed using non-parametric methods. Diagnostic accuracy of biomarkers was determined by area under the curve (AUC) analysis. Results were compared to the relevant literature.

**Results:**

Non-hypoxic origin of cardiac arrest, manual chest compression until ECPR, a short low-flow time until ECPR initiation, low body mass index (BMI) and only a minimal need of extra-corporeal membrane oxygenation support were associated with a good neurological outcome after ECPR. Survivors with good neurological outcome had significantly lower lactate, IL6, D-dimer, and NSE values and demonstrated a rapid decrease in the initial S100 value compared to non-survivors.

**Conclusions:**

A short low-flow time until ECPR initiation is important for a good neurological outcome. Hypoxia-induced cardiac arrest has a high mortality rate even when ECPR and TTM are performed at the emergency scene. ECPR patients with a higher BMI had a worse neurological outcome than patients with a normal BMI. The prognostic biomarkers S100, NSE, lactate, D-dimers and IL6 were reliable indicators of neurological outcome when ECPR and TTM were performed at the emergency scene.

## Background

Out-of-hospital cardiac arrest (OHCA) is the prime cause of mortality in adults in developed industrial countries, with an overall survival rate of 10–30% [1]. The average global incidence of OHCA among adults is 95.9/100,000/year [1–3]. Even in patients with successful return of spontaneous circulation (ROSC), in-hospital survival rates remain extremely low [4]. The underlying mechanisms leading to high mortality and neurological dysfunction in patients who achieve ROSC have been attributed to post-cardiac arrest syndrome (PCAS) [5]. Two pathophysiologies are responsible for PCAS: ischemia and ischemia reperfusion injury (IRI). Ischemia (primary injury) occurs when cardiac arrest (CA) results in an immediate decrease of blood flow followed by a reduction in oxygen delivery. In order to limit ischemic damage, it is necessary to restore circulation as quickly as possible. In addition to the vital importance of starting standard cardiopulmonary resuscitation (CPR) immediately, in cases of prolonged OHCA, extracorporeal cardiopulmonary resuscitation (ECPR) establishes sufficient circulation and oxygen supply at an early stage [6]. Systematic reviews have shown the efficacy of ECPR in OHCA [7–13]. The goal of sufficient resuscitation is to keep the no- and low-flow times as short as possible and thus minimize primary damage [13]. To this aim, our department tries to bring extracorporeal membrane oxygenation (ECMO) to the patient at the emergency scene in order to achieve the shortest possible low-flow time. However, reperfusion after CA is also responsible for a mismatch in oxygen delivery and tissue metabolic rate, while brain injury caused by PCAS is an independent mortality and morbidity factor [5, 14]. Importantly, early and adequate target temperature management (TTM) (mild hypothermia ~ 34.0 °C) after ROSC can lead to improved neurological outcome [2, 13, 15]. The complex cascade of events following brain ischemia is temperature-dependent, meaning hypothermia protects the brain due to a range of mechanisms. Cerebral blood flow, excitotoxicity, lipid peroxidation, production of free radicals, systemic inflammation, activation of the coagulation cascade, and brain swelling are also reduced [16, 17]. A reliable prognostic tool for determining neurological outcome after extracorporeal cardiopulmonary resuscitation in out-of-hospital cardiac arrest (for convenience, this is shortened to ECPR throughout) is indispensable when making further therapeutic decisions for these patients. In other settings, serum biomarkers such as NSE and S100 have been identified as valid predictive markers of neuronal injury [18]. The use of the biomarker S100B protein to determine non-traumatic, traumatic and tumor-associated brain damage is accepted practice [19, 20]. Equally, NSE is recognized as a valid prognostic biomarker for neurological outcome after CA [21]. Hypothermia and veno-arterial extracorporeal membrane oxygenation (VA ECMO) are known to have a potentially negative influence on the predictive value of NSE and S100 biomarkers [22, 23]. Up to now, there have been no studies on whether performing ECPR with TTM (for convenience, this is shortened to ECPR throughout) at the emergency site affects the reliability of neurological outcome biomarkers. The aim of our study was, therefore, to examine the reliability of these biomarkers in prehospital ECPR, reflecting current practice.

## Methods

### Ethical approval

Our institution’s ethics committee approved this study (ethics committee case number: 19-121.56). The need for informed consent to the retrospective collection of anonymized demographics, as well as physiological and hospital outcome data, was waived.

### Study design

A retrospective review of our Regensburg ECMO database identified a total of 69 refractory CA patients treated with out-of-hospital ECPR in addition to conventional advanced cardiac life support (ACLS) between January 2018 and August 2020. Demographic and clinical information was collected retrospectively from our clinical database system, and names and identifying patient numbers were deleted before analysis. All researchers were well-trained in resuscitation and had access to the database without blinding. Because of the retrospective study design, clinicians were not blinded to NSE and S100 measurements. All patients primarily required VA ECMO. Data of patients who died before hospital admission and of patients with incomplete sampling were excluded. Neurological outcome was determined using a cerebral performance category (CPC) score according to which poor neurological outcome was defined as a CPC score ≥ 3, equivalent to severe disability [24].

### Our department’s ECPR indication criteria for infield OHCA

The indication criteria for ECPR in OHCA were as follows: CA was witnessed, basic life support (BLS) had already been initiated by laypeople and ACLS was carried out according to European Resuscitation Council guidelines for less than 60 min until ECPR initiation. ECPR was not initiated in cases where terminal malignancy was known, in cases of traumatic injury with uncontrolled bleeding, in cases of unwitnessed CA or in cases of an extant and credible declaration that the patient did not wish to receive life-prolonging therapies. Patient age was no contraindication. Traumatic CA or controlled bleeding were not included in this study.

### ECPR management in OHCA

In Regensburg, ECPR in OHCA is performed at the emergency site (e.g., the patient's apartment, workplace, or in the street). In order to enable rapid infield ECPR, the ECPR team at the University Hospital Regensburg and the regular ambulance service (AS) are alerted simultaneously. BLS/ACLS treatment in the field is in accordance with the European Resuscitation Council and the American Heart Association guidelines for basic and advanced cardiac life support and post-resuscitation care [2, 13]. After a briefing on the live situation, the decision to perform ECPR is made by the ECPR team. The implantation of ECMO cannulas is performed during ongoing CPR. Both manual and mechanical chest compression were used for CPR in our study. Vascular access for ECPR was achieved by percutaneous dilational cannulation of the femoral vein and arteria, a standard technique for VA ECMO which has been described in detail elsewhere [25, 26]. The ECMO-system CARDIOHELP® (Getinge GmbH, Rastatt, Germany) was used. No arterial blood pressure measurements were taken in the field or during transport to the emergency department. Patients were sedated with midazolam or propofol and fentanyl based on their body weight. Arterial pressure measurement was taken at the emergency department and the first blood sample was taken in the emergency room (ER). The mean arterial pressure (MAP) = diastolic blood pressure + 1/3 (systolic blood pressure – diastolic blood pressure) was set to 50-60 mmHg. Upon arrival at the ER, a patient assessment (whole body computer tomography, cardiac ultrasound, etc.) was performed according to the hospital's internal ER standards. If the cause was not cardiac (e.g., pulmonary artery embolism), guideline-compliant therapy was also given.

#### TTM

All ECPR patients received 2,000 mL of 4–6 °C reperfusion buffer solution administered via ECMO to induce a neuroprotective mild hypothermia (~ 34.0 °C). Because this study is retrospective and neuroprotection via TTM after CA is standard in our department, there is no norm-thermic ECPR group in this study. During transport to the clinic, patients were not actively warmed, though heat retention was assured (blanket etc.). Using a VA ECMO with an integrated heat exchanger, the patient's body temperature was kept at 34.0 °C for 24 h after resuscitation. From hour 25 on, body temperature was raised by 0.5 °C/h to normothermia. From then on, strict avoidance of fever was actively managed.

### Blood sampling and measurements of NSE, S100, lactate, interleukin 6, fibrinogen, d-dimer and platelet count

Serial blood samples were taken from all ECPR patients directly upon arrival at the emergency department. The first sample was collected from the venous line of the ECMO system, and subsequent samples were collected arterially according to our department’s standard blood sampling protocol for ECPR patients. Serum blood samples were collected at 1, 4, 12, 24, 48, 72 and 96 h after VA ECMO implantation. The fully-blinded serum samples were analyzed at the Department of Clinical Chemistry, University Hospital Regensburg. To obtain measurements of S100, IL6 and NSE, a quantitative automated immunoassay (Cobas e411, Roche Diagnostics, Indianapolis, United states) was used. Lactate was measured using quantitative automated photometry (Siemens Dimension Vista 1500, Newark, Germany), fibrinogen was measured using quantitative automated coagulometry (Siemens BCS XP, Newark, Germany) and platelet count was analyzed using flow cytometry (Sysmex XE-5000, Kobe, Japan). All laboratory data were extracted from the clinic’s database system and the ECMO database. Prior to ECPR, no patient had known comorbidities associated with increased NSE or S100 values.

### Neurological outcome parameter

A CPC score was used to assess neurological outcome after ECPR. Patients were allocated to one of three outcome groups: survivors with good neurological outcome (score 1 or 2), survivors with neurological impairment (score 3 or 4), and non-survivors (score 5/brain death) [24]. In this study, we evaluated patient CPC score upon discharge from hospital or—in the case of non-survivors—at the end of therapy. In instances where more than one CPC measurement was performed during hospitalization, only the best CPC score recorded for each respective patient was used for this study.

### Statistical analysis

Serum blood samples were collected 1, 4, 12, 24, 48, 72 and 96 h after ECMO implantation. In cases where a patient’s samples were taken more than once in one of these intervals, the highest value was chosen as characteristic of the patient. In general, the data were not normally distributed. Consequently, all data were depicted and statistically analyzed using non-parametric procedures. The data are presented as medians (25th/75th percentiles) and were statistically analyzed using the Mann–Whitney test. IBM’s SPSS Statistics 25 software (SPSS Inc., Armonk, United States) was used for statistical analysis. P-values < α = 0.05 were considered statistically significant. To evaluate the impact of multiple parameters, the level of significance α was adjusted to α*(k) = 1 − (1 − α)^1/k^ using the error rates method (k = number of paired tests performed) [27]. The ECPR patients were classified according to their survival status as CPC 1–2 survivors, CPC 3–4 survivors, and non-survivors.

## Results

### Demographic characteristics

**Fig. 1:**
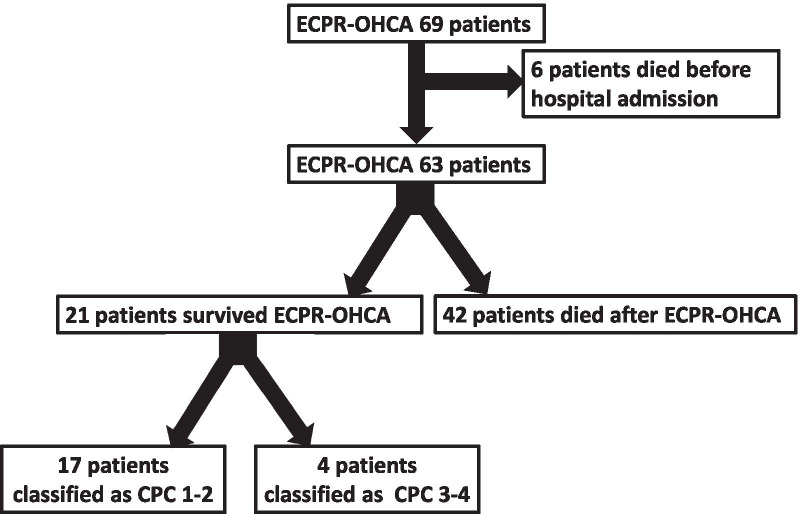
Flow chart of patients treated with ECPR at the emergency scene between January 2018 and August 2020

Between January 2018 and August 2020, infield ECPR was administered to 69 OHCA patients. Six of these 69 patients died before hospital admission or had missing data and were excluded from further analysis (Fig. 1).

**Fig. 2:**
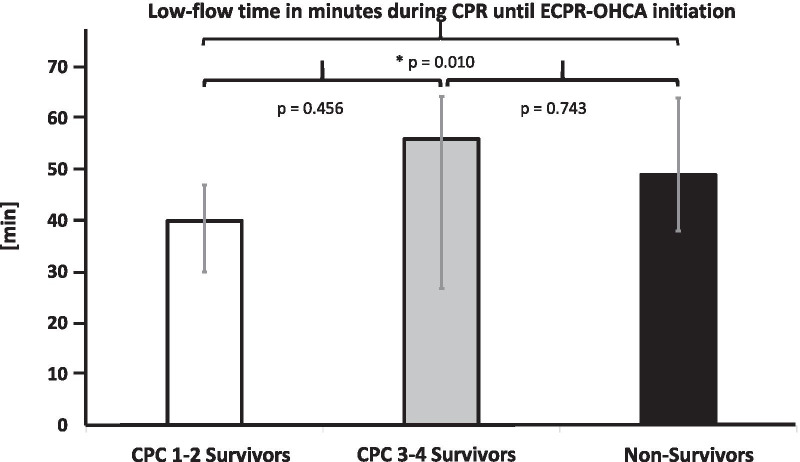
Time Until ECPR Initiation. Low-flow time during CPR until start of ECPR for CPC 1–2 survivors, CPC 3–4 survivors and non-survivors. Values are presented as median and 25th/75th percentiles. * = statistically significant; exact p-values are presented in the figure

73% of the remaining 63 ECPR patients were male. The median age of patients was 58.0 years (48.0 y; 67.0 y), ranging from 13.0 to 82.0 years. In 73% of these patients the primary cause of circulatory arrest was of cardiac origin, whereas 27% of patients needed ACLS due to non-cardiac issues (Table 1). For survivors, the last CPC score was measured upon hospital discharge at a mean of 19 days (range: 4 days to 39 days); for non-survivors, the last CPC score was measured at the end of therapy at a mean of 3 days (range: 1 day to 7 days). A significantly higher proportion of CPC 1–2 survivors had cardiac origin as primary cause of circulatory arrest as compared to non-survivors (88% vs. 64%; p = 0.008). The cause of circulatory arrest (cardiac/non-cardiac) in the CPC 3–4 group was similar to that of the CPC 1–2 and non-survivors groups. Before ECPR was applied, resuscitation was carried out with manual chest compression in 48% of cases and with a mechanical resuscitation device (LUCAS CPR device ®, Redmond, United States) in 52% of cases. In the CPC 1–2 group, significantly more patients underwent manual CPR prior to ECPR (76%) compared to non-survivors (38%), (p = 0.008). CPC 3–4 survivors did not differ from CPC 1–2 or non-survivors. In this study, the mean time before ECPR in OHCA was significantly shorter at 40 min for the CPC 1–2 group (range: 30 min to 47 min)compared to 49 min for non-survivors (range: 38 min; 67 min; p = 0.010). However, time to implantation of ECPR in OHCA did not differ significantly in CPC 3–4 survivors compared to the CPC 1–2 or non-survivor groups (Fig. 2).

**Table 1:** Characteristics of cardiac arrest in OHCA-ECPR patients

	CPC 1–2 survivors	CPC 3–4 survivors	Non-survivors
*Cause of cardiac arrest*			
*Cardiac*	16/17 (94%)	3/4 (75%)	27/42 (64%)
Acute myocardial infarction	7/17 (41%)	2/4 (50%)	23/42
Rhythmogenic event	8/17 (47%)	1/4 (25%)	4/42
Tako Tsubo Cardiomyopathia	1/17 (6%)	Non	Non
*Non-cardiac*	1/17 (6%)	1/4 (25%)	15/42 (36%)
Hypoxia Caused by	1/1 (100%)	Non	8/42 (20%)
Intoxication	1/1	Non	2/42
Drowning	Non	Non	6/42
Acute pulmonary embolism	Non	1/4 (25%)	7/42 (16%)
*Median low-flow time in minutes (25th/75th percentile)*	40 (30; 47)	56 (27; 64)	49 (38; 64)
*Type of CPR until EPCR*			
Manual chest compression	13/17 (76%)	2/4 (50%)	16/42 (38%)
Mechanical resuscitation device	4/17 (24%)	2/4 (50%)	26/42 (62%)
*Mortality of OHCA-ECPR*			
Cardiac origin	27/46 (59%)		
Non-cardiac origin	15/17 (88%)		
*Cause of death in non-survivors*			
Hemodynamic	4/42 (10%)		
Neurological (withdrawal of Life-sustaining therapy in case of cerebral hypoxia)	36/42 (85%)		
Intracerebral bleeding	2/42 (5%)		

Data are presented as absolute count per group / total number of all groups (percentage of the total number). Low-flow time is presented in median and 25th/75th percentiles

After treatment with 4–6 °C resuscitation buffer solution, body temperature was 33.8 °C (32.0 °C; 34.1 °C) upon hospital admission. None of the three groups demonstrated differences in body temperature. After OHCA, patients required ECPR for 3.0 days (2.0 d; 4.0 d), ranging between 1.0 and 18.0 days. The mortality of ECPR patients with non-cardiac cause of circulatory arrest was significantly higher than that of ECPR patients with a cardiac cause of circulatory arrest (88% vs. 59%; p = 0.024) (Table 1).

Survivors and non-survivors demonstrated no differences in age and BMI in our study population. Among survivors, patients with a poor neurological outcome (CPC 3–4) had a significantly higher BMI. Survivors classified as CPC 3–4 needed ECPR for 4.0 days (1.0 d, 3.5 d), which was significantly longer than the 2.0 days (2.0 d, 12.0 d) for CPC 1–2 survivors.

### Proinflammatory cytokines and coagulation markers

**Fig. 3:**
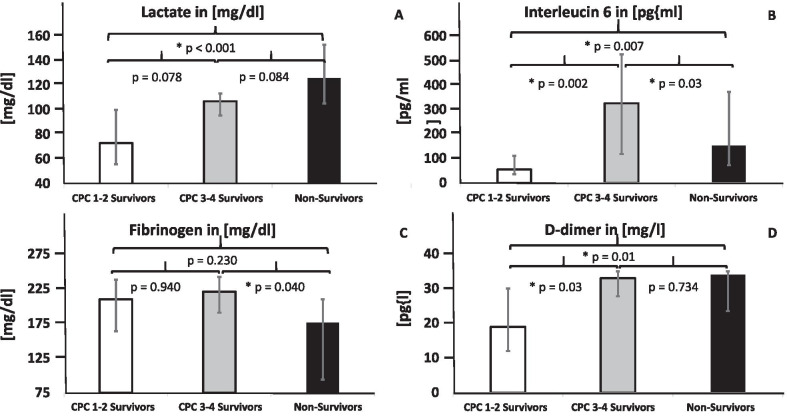
Standard Laboratory Parameters One Hour After ECPR Initiation. **A**–**D** Lactate, IL6, D-dimer, fibrinogen and platelet count one hour after implantation of VA ECMO. Values are presented as median and 25th/75th percentiles. * = statistically significant; exact p-values are presented in the figure. Error rates p-value α*(3) = 0.0179

One hour after ECPR implantation, CPC 1–2 survivors had significantly lower levels of lactate, interleukin 6 (IL6) and D-dimer than non-survivors (Fig. 3A, B). Fibrinogen values were significantly higher in the CPC 1–2 survivors group compared to non-survivors (Fig. 3B).

All data for lactate, IL6, fibrinogen and D-dimer are presented in Table 2. There were no differences in the platelet counts of CPC 1–2 survivors, CPC 3–4 survivors and non-survivors at one hour after ECPR implantation.

**Table 2:** Lactate, IL6, D-dimer, fibrinogen and platelet count

	CPC 1–2 survivors	CPC 3–4 survivors	Non-survivors	p values
Lactate [mg/dl]	73.0 (56.0; 100.0)	107.0 (95.5; 113.3)	126 (105.3; 153.0)	see Fig. 3
IL6 [pg/ml]	55.0 (36.0; 111.0)	326.0 (118.3; 526.3)	153.0 (72.5; 372.5)	see Fig. 3
D-dimer [mg/l]	19.0 (12.0; 30.0)	33.0 (27.8; 35.0)	34.0 (23.5; 35.0)	see Fig. 3
Fibrinogen [mg/dl]	210.0 (238.0; 164.0)	221.0 (190.5; 242.3)	176.0 (94.0; 210.0)	see Fig. 3
Platelet count [/nl]	157.0 (129.0; 219.0)	164.0 (126.0; 203.0)	154.0 (102.5; 187.0)	n.s.

Lactate, IL6, D-dimer, fibrinogen and platelet count one hour after ECPR implantation. Data are presented in median and 25th/75th percentiles; p-values are presented in Fig. 3. p < 0.050 rated as significant; n.s. = not significant

#### S100

**Fig. 4:**
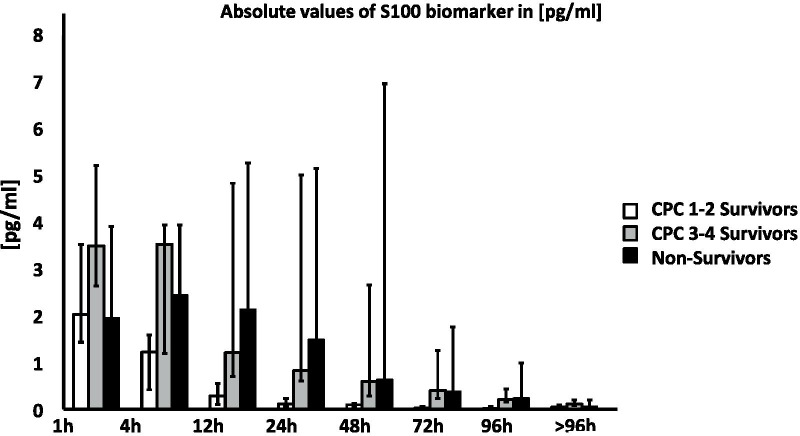
Neurological outcome biomarker S100. Absolute values of S100 measured in plasma in [pg/ml] 1–96 h post-ECPR implantation. Data are presented as medians and 25th/75th percentiles

In all groups, increased S100 values were observed in the first hour following VA ECMO implantation when compared to a healthy control group from the literature [20, 28]. Statistical analysis showed that absolute concentrations ​​of the S100 protein scattered significantly over the entire observation period (Fig. 4).

**Fig. 5:**
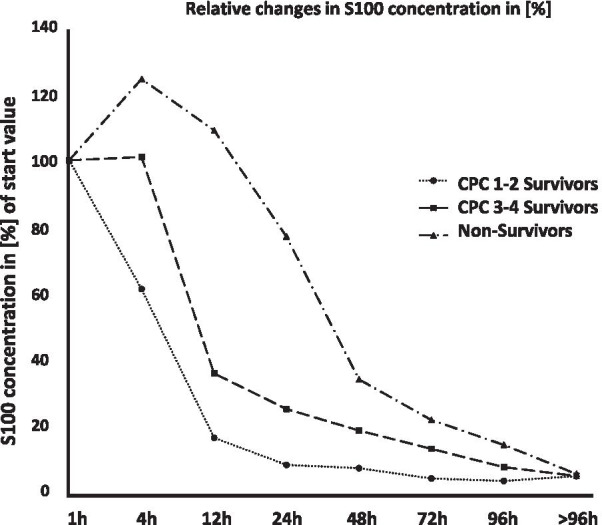
Relative changes in neurological outcome biomarker S100. Relative changes in S100 concentration 1–96 h post-ECPR. Change in S100 over time as percentage. 1 h data of each outcome group normalized to 100%

With the intention of analyzing the change in S100, the first S100 value measured at 100% was determined and then subsequent values were compared in relation to the first value (Fig. 5). Decreasing S100 values were observed in the CPC 1–2 survivor group, beginning immediately after the first measurement (Fig. 5).

S100 values halved in the CPC 1–2 survivors group at each successive time point over the first 48 h. The decrease in S100 values in the survivor group with impaired neurological outcome (CPC 3–4) began after a delay of 4 to 12 h after VA ECMO initiation. In the non-survivor group, S100 values initially increased and then decreased after 12 h, though the reduction in concentration was less distinct until 24 h had elapsed. For this group, a halving of the start values between time points was only observed beyond 48 h.

### NSE

**Fig. 6:**
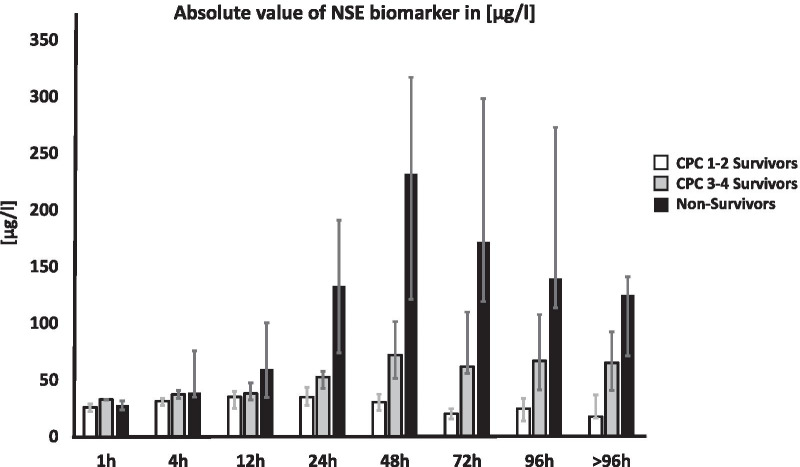
Neurological outcome biomarker NSE. Time course of NSE biomarker 1–96 h post-ECPR implantation. Data are presented as medians and 25th/75th percentiles. Exact p-values are presented in Table 3

While the interference of hemolysis on S100 measurements was relatively low, NSE analysis was greatly complicated by hemolysis. Interference due to hemolysis was found in 67% of all NSE measurements carried out in the first hour. This interference decreased to 51% and then 22% over the next two measurements, which were taken at 4 h and 12 h, respectively. At 24 h after implantation the interference values were similar to that of the S100 measurements (approximately 10%). No statistically significant difference in NSE could be detected at either 1 h or 4 h after ECPR implantation (Fig. 6).

At 12 h after ECPR initiation the non-survivors had a significantly higher median NSE level compared to survivors in the CPC 1–2 group (59 pg/L vs. 35 pg/L; p = 0.028). NSE concentrations in CPC 3–4 survivors and non-survivors were not significantly different. At 24 h post-ECPR implantation, CPC 1–2 survivor group patients had a median NSE level of 35 pg/L. This was significantly lower than that of patients in the non-survivor group (133 pg/L; p < 0.001). The highest NSE values were measured 48 h after ECPR implantation, and non-survivors had the highest NSE values of all groups in our study. The NSE median was 232 pg/L for non-survivors at 48 h; 75% of all non-survivors had an NSE value > 120 pg/L 48 h after ECPR initiation. In contrast, the median NSE value at 48 h was 31 pg/L for CPC 1–2 survivors, 75% of whom had an NSE (48 h) value < 38 pg/L at this time. In the CPC 3–4 survivor group, the median NSE value at 48 h was 72 pg/L and 75% of CPC 3–4 survivors had an NSE value < 101 pg/L. The values of all three groups at 48 h post-ECPR demonstrated statistically significant differences. All exact p-values in these instances of significant difference are presented in Table 3. After the 48-h measurement, NSE values of CPC 1–2 survivors and those of non-survivors decreased to approximately half of the maximum levels in the following 96 h.

**Table 3:** p value of NSE values (× h) post-ECMO

Interval (h)	CPC 1–2 survivors vs. CPC 3–4 survivors	CPC 1–2 survivors vs. non-survivors	CPC 3–4 survivors vs. non-survivors
1	0.025*	0.524	0.066
4	0.143	0.048*	0.770
12	0.337	0.028*	0.096
24	0.065	< 0.001*	0.002*
48	0.002*	< 0.001*	< 0.001*
72	< 0.001*	< 0.001*	0.006*
96	0.008*	< 0.001*	0.009*
> 96	0.032*	0.007*	0.123

Exact p-value comparison of survivor group NSE values for each measurement interval; p < 0.050 was rated as significant and marked with an *. Error rates p-value α*(3) = 0.0179

**Fig. 7:**
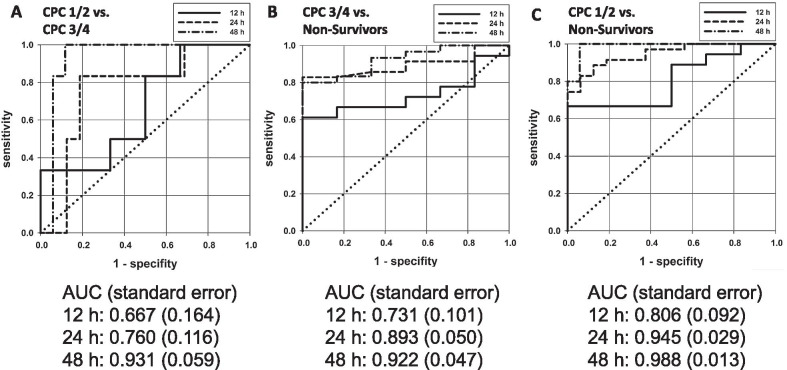
ROC curves of neurological biomarker NSE. ROC curves of NSE values. Individual comparison groups: **A** CPC 1–2 survivors vs. CPC 3–4 survivors; **B** CPC 3–4 survivors vs. non-survivors; **C** CPC 1–2 survivors vs. non-survivors. Individual examination periods (12 h, 24 h and 48 h) are plotted in one figure. AUC = area under the curve

Receiver operating characteristic (ROC) curves visually represent the relationship between efficiency and the error rate for various parameter values. The ROC analyses in our study clearly showed that NSE values were not valid indicators of a patient’s chance of survival or neurological outcome in the first 12 h after ECPR. However, after 24 h it was possible to determine ECPR survivors based on NSE values. An assessment of neurological outcome was possible after 48 h at the earliest (Fig. 7A–C).

## Discussion

The main finding of our study is that S100 and NSE values were reliable biomarkers in determining the neurological outcome of survivors who received ECPR at the emergency scene. A relative decrease in S100 during the first 24 h indicated a good neurological outcome, while an increase in S100 to base level in the first 24 h post-cannulation was associated with a worse neurological outcome. In our study, S100 values were determined in 88% of all cases in the first 12 h after OHCA – despite hemolysis. The wide range of S100 biomarker values in our study meant that it was not possible to determine a cut-off value for neurological outcome after ECPR. This wide range is possibly due to the fact that S100 is expressed in different tissues: in addition to neuronal tissue, S100 is expressed in muscle and fat tissue and helps to regulate many intra- and extracellular processes [29]. Consequently, fluctuations in S100 values can be interpreted as an interindividual reaction to PCAS stress manifesting in different tissues [28]. However, even if the absolute S100 value cannot be used as a neurological prognostic parameter, the rapid drop in S100 concentrations within the first 24 h after ECPR can predict neurological outcome. In our study, a good neurological outcome was associated with a continual decrease in S100 relative to the first value (measured within 12 h). A delayed decrease in S100 or even an increase at 4 and 12 h after ECPR was associated with a poor neurological outcome. These findings were in line with the results from other studies investigating changes in S100B after CA [20, 30]; it is accepted practice to use the S100B protein biomarker to determine non-traumatic, traumatic and tumor-associated brain damage [19]. Because no valid median/ cut-off value could be determined in our study due to the large spread of the S100 values, S100 has a lower prognostic validity in neurological outcome after CA and ECPR compared to NSE. The sole decision about the neurological outcome according to CA and ECPR based on the S100 course does not seem to be reliable for the reasons mentioned above. In combination with other biomarkers, the S100 course can improve the prognostic validity of the neurological out come after CA and ECPR.

Our study also found that NSE was a good predictor for survival in ECPR. However, obtaining NSE measurements was greatly impaired by hemolysis in the first 12 h after OHCA [22]. This may explain the failure to identify significant differences in NSE values in the first 12 h after ECPR. As the interference of hemolysis decreased, however, patient outcome could be determined based on SE values: at 24 h post-ECPR, survivors could be distinguished from non-survivors; at 48 h post-ECPR, survivors with a good neurological outcome could be distinguished from those with a poor neurological outcome. These findings are shown in the ROC curves (Fig. 7A–C). In our study, 75% of all patients who did not exceed an NSE value of 58 pg/L 24 h after ECPR survived. Furthermore, 75% of all patients did not survive an NSE value of more than 74 pg/L, while 75% of all patients who did not exceed an NSE value of 38 pg/L after 48 h survived ECPR with a good neurological outcome (CPC 1–2). The cut-off value of NSE used to determine survivors and non-survivors in OHCA with ECPR was comparable to that used in other OHCA studies without ECPR (69 pg/L) [31] (76 pg/L) [32].

Lactate and D-dimer were the earliest reliable prognostic markers for survival in ECPR in our study. At one hour after ECPR, non-survivors had a significantly increased lactate value compared to CPC 1–2 survivors. This finding is comparable to other studies without ECPR [39]. However, it was not possible to differentiate between CPC 1–2 and CPC 3–4 survivors based on the 1-h lactate value in our study. Non-survivors had increased coagulation activity (D-dimer) and inflammatory response (IL6) compared to survivors (CPC 1–2). Consumption of coagulation factors, paired with an inflammatory reaction, could indicate that disseminated intravascular coagulopathy was the cause of neuronal tissue damage due to microvascular damage [40]. It is noteworthy that these changes in coagulation parameters already begin to occur in the first hour after OHCA and are thus useful when making a prognostic estimation of mortality. In our study, an increase in D-dimers and a decrease in fibrinogen paired with an increase in IL6 were associated with significantly higher mortality.

The demographic data of our ECPR study population are similar to those of other OHCA studies in terms of age, sex and CA cause [33–35]. In our study, however, ECPR patients with cardiac origin had a lower mortality rate (59%) than those from the literature (67–92%) [34, 35]. ECPR patients with non-cardiac origin (e.g. near-drowning) had a similar mortality rate in both our study and the literature (88%) [12, 34]. Similar findings were reported by M. Kuisma et al. [36]. In their study, they proposed that the lower survival rate in non-cardiac OHCA was due to a lower frequency of bystander CPR in these cases [36]. As our study only included patients with witnessed CA who were provided with BLS through bystander CPR, Kuisma et al.’s explanation cannot be the main cause of the non-cardiac origin group’s lower survival rate in our study. In addition, Kuisma et al. reported that the main causes of non-cardiac OHCA were drowning, pulmonary embolism and intoxication. These were also the most common causes of non-cardiac resuscitation in our study, with the resulting hypoxia as the cause of OHCA. In 85% of all cases in this study, hypoxic brain damage was the cause of death in ECPR. It is therefore our opinion that hypoxia prior to OHCA is the leading mortality factor. It is, however, not the only factor that influences the outcome of ECPR.

In our study, ECPR patients with an elevated BMI often had a poor neurological outcome compared to patients with a normal BMI. This is because obesity renders sufficient chest compression and ventilation during resuscitation more difficult [37]. It is also known that obesity increases oxygen consumption (VO2) while lowering the body’s oxygen reserves [38]. All of these factors result in a higher rate of hypoxia in obese people. The pre-existing oxygen deficiency caused by obesity is comparable to hypoxia in non-cardiac origin CA, which in turn increases ischemia/reperfusion syndrome, thus worsening the outcome after OHCA [5]. Furthermore, chronic inflammation in obesity has an additional negative impact on survival after CA [39].

In addition, a shorter length of CPR before ECPR initiation was associated with a significantly higher survival rate combined with a good neurological outcome. The findings in our study are similar to those in other studies in which a short low-flow time is key to surviving OHCA [40]. In our study, a time period of more than 60 min until initiating ECPR was directly linked to lower survival rates. These findings were similar to those of a study by Takayuki Otani et al. [40].

In our study, manual chest compression was associated with a good neurological outcome compared to the use of mechanical resuscitation devices; our findings were that mechanical resuscitation devices were not superior to manual chest compression. In our opinion, it was not the use of mechanical resuscitation devices but rather the longer low-flow time prior to ECPR initiation that influenced the ECPR outcome. As mechanical resuscitation devices are often used in long-term CPR, this observation highlights that a short low-flow time is key to a successful resuscitation, even with ECPR. Whether injury due to mechanical resuscitation device use impacted survival was not subject to investigation in this study.

The prolonged need of ECMO support was associated with a poor neurological outcome in our study. ECPR in OHCA is a maximally invasive therapy; it is also associated with complications. The longer a patient needs ECMO support, the easier it is for an outcome-impairing event to occur (bleeding, systemic inflammatory response syndromes, and infections) [41, 42]. Only needing ECMO for a short period is therefore associated with a better outcome.

The data presented in our study should be interpreted within the constraints of some potential limitations. The majority of the patients included in our study were older and male. It is therefore not possible to make a reliable, gender and age independent statement about the outcome of ECPR performed at the emergency scene with data from this study. When interpreting the data, it should also be noted that the group sizes differ due to the retrospective nature of our study. Finally, the limited number of patients included in the study should be mentioned as a limitation.

Methodological limitations must also be taken into account when interpreting our study. In clinical practice, it was not always possible to collect blood at strictly regular intervals after ECPR. For this reason, we had to define larger sampling times in our study. Due to the nature of such an emergency procedure the times of collection varied and are grouped as follows: samples ≤ 2 h = 1 h; samples > 2 h ≤ 6 h = 4 h; samples > 6 h ≤ 12 h = 12 h; samples > 12 h ≤ 30 h = 30 h; samples > 30 h ≤ 55 h = 48 h; samples > 55 h ≤ 78 h = 72 h; samples > 78 h ≤ 100 h = 96 h; samples > 100 h =  > 96 h. As a result, the precise time point of blood sample collection may have varied within these intervals, which could affect the results. Due to the retrospective nature of this study, incorrect measurements (e.g., due to hemolysis) could not be taken again. However, as influences on the blood samples—such as hemolysis and incorrect measurements—were random we have not included these as a bias.

Due to the retrospective design of this study clinicians were not blinded to NSE and S100 measurements. In our clinic there is, in fact, no cut-off value for these parameters, no cut-off is there for therapy limitation. Though unblinding would normally be a major limitation, we argue that its influence was negligible as the study was carried out in four different intensive care units (ICUs) with different clinicians.

Treating CA with ECPR is a complex process in which a large number of complications can occur (injury to large vessels, major bleeding, leg ischemia, etc.). It is conceivable that such complications could also cause hemolysis, among other conditions/disorders/complaints, and thus affect the laboratory parameters of this study.

Due to the fact that ECPR is superior to standard ACLS therapy in survival in our opinion a prospective randomized study design for ECPR therapy in case of CA is not justifiable from an ethical point of view [43]. Therefore, the only way to counter the limitations in our study is to increase the number of cases. It is the aim of a future investigation to include a larger number of ECPR patients in a multicentre study in order to validate the findings of this study.

## Conclusions

Hypoxia-induced OHCA, as well as an increased BMI were associated with poor survival and neurological outcome, even with ECPR. A short low-flow time until ECPR initiation (< 60 min) was important for survival and a good neurological outcome. Lactate and D-dimer were the earliest reliable prognostic markers for survival in ECPR. In addition, NSE was a good predictor for survival in OHCA-ECPR at the emergency scene. NSE values was a reliable biomarker in determining the neurological outcome of survivors in ECPR at the emergency scene. A combination of NSE and S100 can improve the prognostic validity for neurological outcome after CA and ECPR. This is a promising field of research which merits further investigation due to its potential impact on treatment and patient outcome in ECPR.

## Data Availability

The datasets used and analyzed during the current study are available from the corresponding author on reasonable request.

## Abbreviations

ACLS: Advanced cardiac life support
BLS: Basic life support
BMI: Body mass index
CA: Cardiac arrest
CT: Computed tomography
CPC: Cerebral performance category
CPR: Cardiopulmonary resuscitation
°C: Degree(s) Celsius
d: Day
dL: Deciliters
ECPR: Extracorporeal cardiopulmonary resuscitation
ECMO: Extracorporeal membrane oxygenation
e.g.: For example
ER: Emergency room
h: Hours
HD: Haemodynamic
ICU: Intensive care unit
IL6: Interleukin 6
IRI: Ischemia reperfusion injury
L: Liters
NSE: Neuron-specific enolase
nL: Nanoliters
ns: Not significant
MAP: Mean arterial pressure
mg: Milligrams
min: Minutes
mL: Milliliters
mmHg: Millimeter quicksilver
OHCA: Out-of-hospital cardiac arrest
PCAS: Post-cardiac arrest syndrome
pg: Picograms
ROSC: Return of spontaneous circulation
ROC: Receiver operating characteristic
S100: Calcium binding protein
TTM: Target temperature management
VA ECMO: Veno-arterial extracorporeal membrane oxygenation
y: Year
